# Effects of Training on Cardiorespiratory Fitness in Triathletes: A Systematic Review and Meta-Analysis

**DOI:** 10.3390/ijerph182413332

**Published:** 2021-12-17

**Authors:** Alicia Borrego-Sánchez, Maria Jesus Vinolo-Gil, Maria de-la-Casa-Almeida, Manuel Rodríguez-Huguet, María Jesús Casuso-Holgado, Rocío Martín-Valero

**Affiliations:** 1 Department of Physiotherapy, Faculty of Health Science, Ampliacion de Campus de Teatinos, University of Malaga, C/Arquitecto Francisco Peñalosa 3, 29071 Malaga, Spain; aliciaborrego98@gmail.com; 2 Department of Nursing and Physiotherapy, Faculty of Nursing and Physiotherapy, University of Cadiz, 11009 Cadiz, Spain; mariajesus.vinolo@uca.es (M.J.V.-G.); manuel.rodriguez@uca.es (M.R.-H.); 3 CMU Rehabilitation Intercentres-Interlevels Puerto Real and Cadiz Hospitals, Cádiz-La Janda Health District, 11006 Cadiz, Spain; 4 Department of Physiotherapy, Faculty of Nursing, Physiotherapy and Podiatry, University of Seville, 41009 Seville, Spain; mcasa@us.es (M.d.-l.-C.-A.); mcasuso@us.es (M.J.C.-H.)

**Keywords:** oxygen consumption, physical endurance, anaerobic thresholds, cardiorespiratory fitness

## Abstract

Triathlon is an aerobic sport, which is commonly measured by maximal aerobic consumption (VO_2_max). Objective: to analyze the changes produced in cardiorespiratory and physiological measurements during practice, which determine triathletes’ performance level. A systematic review and a meta-analysis based on PRISMA protocol and registered in PROSPERO (CRD42020189076) was conducted. The research was performed using PubMed, SPORTDiscus, Embase, Dialnet, Web of Science (WOS) and MEDLINE databases during February and March 2020. Studies that measured cardiorespiratory variables in triathletes published in the last 10 years were included. Results: 713 articles were identified, with 25 studies selected for the systematic review and five articles for the meta-analysis. These articles concluded that the main cardiorespiratory variables that determine triathletes’ performance were modified depending on the triathlon segment performed and the athletes’ sex and age. The meta-analysis showed no conclusive results related to the effects of changes in VO_2_max in triathletes’ performance [SMD = −0.21; 95%CI: (−0.84 to 0.43)]. Conclusions: cardiorespiratory fitness, in terms of VO_2_max and ventilatory thresholds, is the strongest predictor of performance in triathlon. This response may be affected depending on the triathlon segment performed and the athlete’s age or sex, leading to both physiological and biomechanical alterations that affect competition performance.

## 1. Introduction

Triathlon is an endurance sport characterized by combining three different sport disciplines, which are swimming, cycling, and running, performed in that order without stopping the chronometer during the competition. The period of time in which the triathlete changes from one sport discipline to another is called transition (T). There are two transitions: one between swimming and cycling (T1) and another between cycling and running (T2) [1]. This sport emerged in the late 70s and, since then, it has been increasing in popularity and developing to what we know today. Thus, there are many triathlon formats depending on the overall distance performed (Table 1). In this study, we will only focus on sprint, Olympic, half-Ironman, and Ironman™ distances [2].

The main goal of triathlon is to finish the competition as quickly as possible. As a result, the athlete must have a suitable aerobic endurance that allows him/her to keep an appropriate performance during the race [3]. In this way, it is essential to identify which factors are the most influential in triathletes’ performance and aerobic endurance, and how they are modified due to sports practice [4]. The most common measures to determine the degree of triathletes’ aerobic endurance are maximal oxygen consumption (VO_2_max) and ventilatory thresholds (VTs). Additionally, there are other parameters that evaluate athletes’ aerobic endurance, such as blood lactate concentration (LT), running economy (RE), and heart rate (HR) [5,6]. Furthermore, these factors tend to be modified depending on age and sex, so these two variables should also be considered [7,8].

The objective of this systematic review and meta-analysis is to investigate the changes produced in cardiorespiratory fitness during sports practice that determine the level of triathletes’ performance, analyzing the differences depending on age, sex, training level, and competitive distance. The secondary objective was to propose a training program based on the results obtained that may improve triathletes’ performance during competitions.

## 2. Materials and Methods

The present systematic review and meta-analysis was conducted following the PRISMA (Preferred Reporting Items for Systematic Reviews and Meta-Analyses) recommendations (http://www.prisma-statement.org, accessed on 1 February 2020) [9]. The register number in the International Prospective Register of Systematic Review (PROSPERO) is CRD42020189076.

### 2.1. Search Strategy

The bibliographical research was carried out using the Pubmed, SPORTDiscus, Embase, Dialnet, Web of Science (WOS), and MEDLINE databases, focusing on those articles that measured cardiorespiratory variables (VO_2_max and ventilatory thresholds mainly) in order to determine the physiological changes produced in cardiorespiratory fitness of athletes who practice triathlon. The database research was complemented with a manual review of the reference lists of relevant studies. This research was investigated during February and March 2020. The search strategy was performed following the PICO model (Population, Intervention, Comparison, Outcome), combining the terms chosen (triathlon, VO_2_max, ventilatory thresholds, cardiorespiratory fitness) with different Boolean operators [9]. The search range was reduced to articles published in the last 10 years to show a current panorama of the field of study. Titles and abstracts of retrieved articles were individually evaluated by two reviewers (A.B.S. and R.M.V.) to assess their eligibility for review and meta-analysis. Authors of articles not published in open access were contacted when possible. In case of doubt, authors resolved disagreements by consensus and consulted a third author (M.J.V.G.) when necessary. The search strategy process of the articles selected for this review can be found in Table 2.

### 2.2. Selection Criteria

Studies that included the following criteria were selected: (1) adult participants (mean age ≥15 years old) who practiced conventional triathlon, elite or non-elite, from male or female category, and who competed in sprint, Olympic, half-Ironman, and/or Ironman distances; (2) participants without any pathological condition or previous injury; (3) articles that evaluated physiological variables related to cardiorespiratory fitness; (4) articles classified as meta-analysis, reviews, randomized/non-randomized controlled trials, cohort studies, and cross-sectional studies; (5) articles published in English or Spanish. Studies were excluded if: (1) participants mean age was under 15 years old; (2) cardiorespiratory variables were not studied; (3) they were focused on duathlon, cross triathlon, or any other conventional triathlon event than sprint, Olympic, half-Ironman, or Ironman distances; (4) the study’s full text access were not free.

### 2.3. Quality Assessment

The evaluation of the methodological quality of the studies selected in this systematic review and meta-analysis was performed using the Physiotherapy Evidence Database (PEDro) scale. This scale is made up of 11 criteria which assesses the internal validity of articles [10,11,12]. To assess the methodological quality of the reviews selected for the present study, the Measurement Tool to Assess Systematic Reviews (AMSTAR-2) questionnaire was used. This tool consists of a questionnaire of 16 domains, where each one can be scored as “yes” when the item is met, “no” when the item is not met or is not reported in the review, and “partial-yes” if the item is incompletely fulfilled. Depending on the answers obtained, each review will present a specific level of confidence, which can be high, moderate, low, or critically low [13,14].

### 2.4. Risk of Bias of Included Studies

The risk of bias was calculated for each study selected using the Cochrane Collaboration Tool [15]. The following types of bias were assessed: selection bias, performance bias, detection bias, attrition bias, reporting bias, and other bias. Two reviewers (A.B.S. and R.M.V.) assessed the methodological quality and the risk of bias of the studies. In case of doubt, authors resolved disagreements by consensus and consulting a third author (M.J.V.G.) when necessary.

### 2.5. Data Extraction and Statistical Analysis

Data extraction was carried out by two investigators (A.B.S. and R.M.V.) for each article chosen for the present study. In case of doubt, authors resolved disagreements by consensus and consulting a third author (M.J.V.G.) when necessary. A meta-analysis was performed on the studies that met the inclusion criteria and used the same outcome measure. Thus, from each study, data on the sample size and the mean value and standard deviation of the maximal oxygen consumption (VO_2_max) of the pre-intervention and post-intervention, both for the experimental group (EX) and the control group (CON), were collected. VO_2_max was expressed in relative values (mL/kg/min). To calculate the effect size (ES), the standardized mean difference (SMD) was used with a 95% confidence interval (95% CI) and a statistical significance set at *p* < 0.05. The heterogeneity of the effect sizes of the studies was determined using the I^2^ statistic. To that end, a random-effects model was used. The results of the included studies are shown in a forest plot [16,17,18]. The statistical analysis was carried out with the statistical software Review Manager (RevMan) [Computer program]. Version 5.4.1, Copenhagen, Denmark, The Cochrane Collaboration, 2020.

For the analysis, each study provided data on two intervention groups formed by triathletes, except for one of the studies that was composed of one group of triathletes and another group of soccer players [19]. In addition, one of the studies included was broken up in two parts because it included two different interventions, one of them consisting of a cycling test and the other one related to a running test [20].

## 3. Results

### 3.1. Study Selection

**Figure 1:**
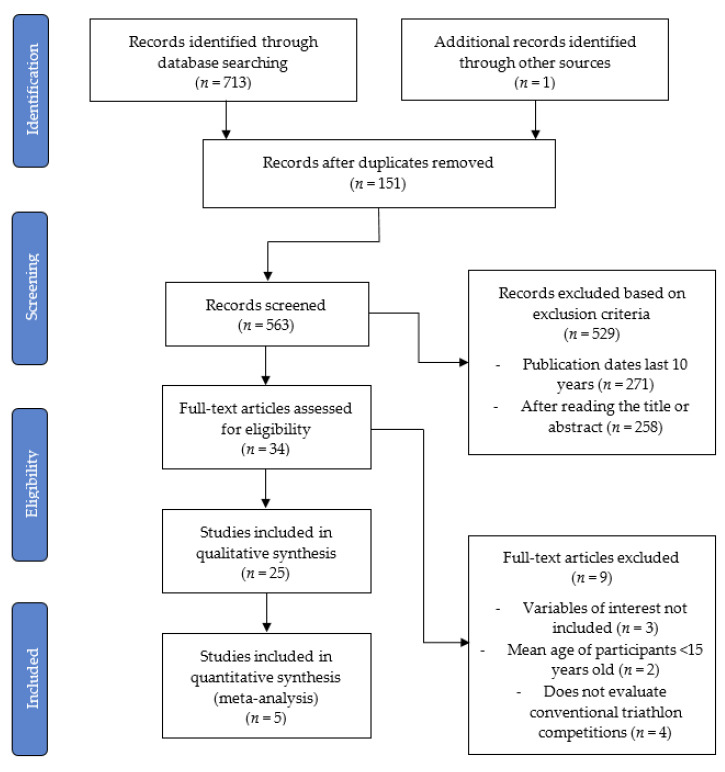
PRISMA flow diagram of the study selection process.

In the search strategy, a total of 713 articles were found (Figure 1). From the total number of articles, 25 studies were selected for the systematic review, of which five articles were included in the meta-analysis. Regarding the study design of the articles selected for the systematic review, nine reviews, one randomized controlled trial (RCT), 13 cohort studies and two cross-sectional studies were found. A summary of the study selection process can be observed in Figure 1.

### 3.2. Methodological Quality of Included Studies

Table 3 shows the final grade obtained by the articles selected after reviewing the methodological quality using the PEDro scale. According to the PEDro scale, two of the 16 articles reviewed achieved a score of 7, which is considered Level 1 evidence (good, 12.5%, 2/16), four studies had a score of 5, which is considered Level 2 evidence (acceptable, 25%, 4/16); and ten studies obtained a score equal to or less than 4, which indicates Level 3 evidence (poor, 62.5%, 10/16). Therefore, 37.5% of the articles reviewed achieved a moderate methodological quality and a low risk of bias.

Similarly, Table 4 shows the rating of the methodological quality of the reviews included in the present study using the AMSTAR-2 scale. In it, Items 11 and 12 were not counted for the final score (indicated with the symbol “–”), because none of the articles included carried out a meta-analysis. It was observed that all of the reviews showed weaknesses in most of the domains considered critical. These weaknesses were related to the lack of registration of a prior protocol of the conducting reviews (Item 2), the lack of suitable bibliographical research (Item 4), the absence of justification of the articles excluded (Item 7), the evaluation and risk of bias of the articles included (Items 9 and 13), and the evaluation and discussion of the impact of publication bias on the results obtained (Item 15). Therefore, the methodological quality of the reviews selected was considered critically low [13].

### 3.3. Risk of Bias of Included Studies

**Figure 2:**
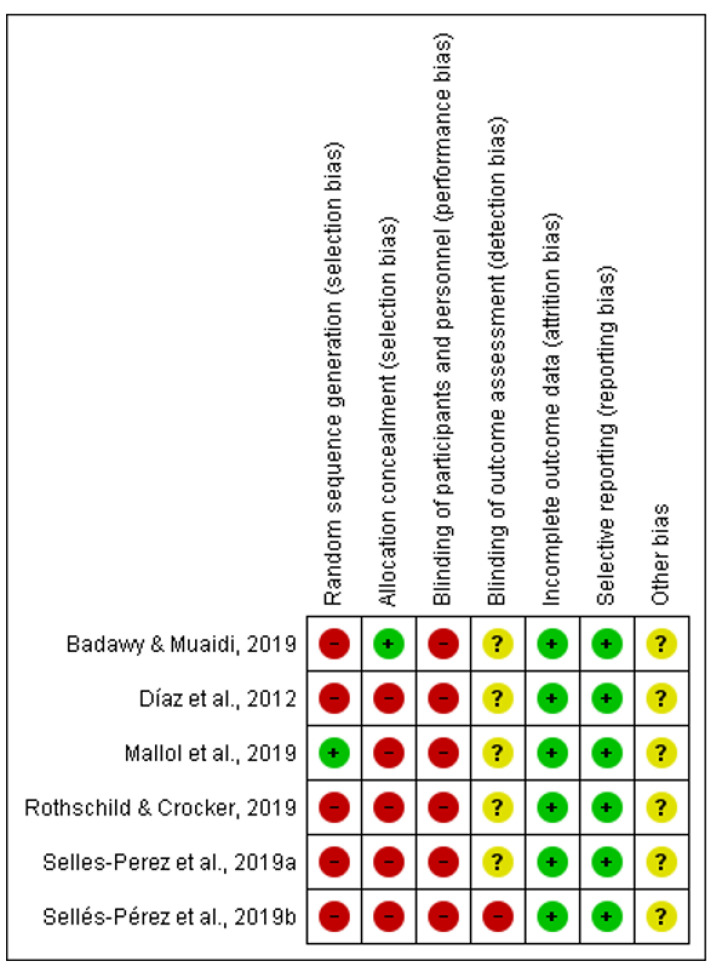
Risk of bias summary [6,19,20,21,24,26].

**Figure 3:**
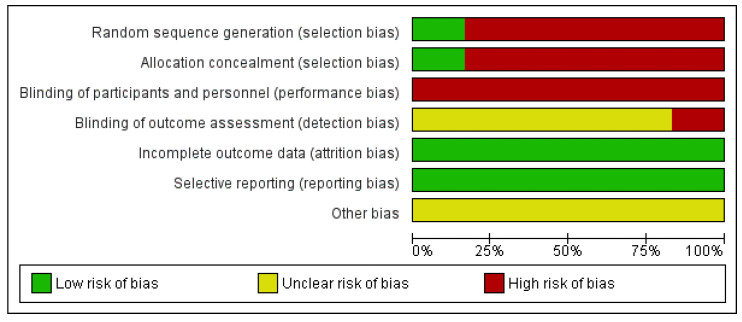
Risk of bias graph.

The Cochrane Risk of Bias Assessment Tool [15] was used to assess the risk of bias of the articles included in this review. The results of the risk of bias can be observed in Figure 2. It should be noted that the risk of bias is high in relation to selection bias because there was randomization without allocation concealment only in one of the articles [19]. With respect to attrition bias and reporting bias, all of the them were low risk (Figure 3).

### 3.4. Study Design and Intervention Characteristics

Regarding the main characteristics of the different studies (Table 5), it can be seen that a total of 884 subjects were evaluated in the studies selected. The article that used the largest sample size was Badawy & Muaidi, 2019 [19], with 22 subjects, while the one that used the smallest sample size was Walsh et al., 2015 [29], with six participants. With regards to the characteristics of the participants, all of them were older than 18 years old, except in one study that also analyzed athletes aged 15–16 years old [21]. In addition, the study population was mainly made up of male athletes. Only three investigations evaluated women, even though the number of women was lower than the number of men [6,22,26]. Furthermore, with respect to training level, most of the studies analyzed recreational-level triathletes; while only four of them evaluated elite athletes [19,21,29,31].

According to the intervention carried out (Table 5), the studies could be classified into four groups: studies that evaluated the impact of the execution of a triathlon segment on the triathletes’ subsequent performance [3,6,21,22,27,29,31], different training programs for triathletes [1,20,24,26,28], physiological changes produced in triathletes due to their age [23], and triathletes’ physiological variables compared to other sports [19]. These interventions had a mean duration of two and six sessions [3,6,19,22,23,27,28]. In Table 6, the different study variables analyzed can be observed. Thus, the most evaluated variables were VO_2_max, ventilatory thresholds, heart rate, and anthropometric and perceptual measurements. The evaluations mainly used maximal and submaximal exercise stress tests with gas analyzers [1,6,19,20,21,23,24,26,27,29,31], indirect calorimetry [3,28], or near infrared spectroscopy (NIRS) [22], as well as heart rate monitors.

Regarding the results of the articles selected, they can be classified according to the intervention carried out:

- Effects of triathlon depending on the characteristics of the athlete:

In relation to sex, gender differences in triathlon performance has decreased in the last three decades, currently representing differences of 12–18% in recreational-level triathletes and 10–12% in elite triathletes [8]. High levels of VO_2_max have been observed in triathletes, which is associated with high levels of aerobic power. In male elite triathletes, VO_2_max levels were higher than 80 mL/kg/min, while in female elite triathletes they were greater than 70 mL/kg/min. However, these values were lower in recreational-level triathletes [4,5,7]. Additionally, no differences were observed in anaerobic thresholds and in running economy between both sexes [2,8].

In regards to age, only two studies evaluated master (55–70 years old) and young (20–35 years old) triathletes’ performance to determine the changes produced in these athletes due to their age [23,25]. In this way, significantly lower VO_2_max, ventilatory thresholds, and locomotor efficiency values were observed in master triathletes, as well as higher body fat levels [25]. However, non-significant differences were found in the strength parameters between both groups [23].

- Effects of a triathlon segment execution on the subsequent performance during the competition.

These studies determined that the execution of a previous cycling segment adversely affects triathletes’ performance on subsequent running [3,21,22,27,29,31], as the swimming segment affects subsequent cycling performance [6]. To that end, some articles analyzed different parameters during running tests performed after previous cycling and isolated running tests execution [3,21,22,27,29,31]. Significant increases were reported in certain cardiorespiratory variables, such as minute ventilation (VE) [21,27,31], ventilatory equivalents for oxygen (VE/VO_2_) [21] and carbon dioxide (VE/VCO_2_) [21,31], oxygen consumption (VO_2_) [27,29,31], respiratory exchange ratio (RER) and respiratory rate (RR) [31], heart rate (HR) [3,21,27,29,31], and blood lactate concentration [3,27,31].

In addition, in an article that studied running performance in moderately trained triathletes, significant increases in VE, HR, and blood lactate concentration were observed during the race when running was performed after one hour of cycling [3,27]. Furthermore, these changes were greater when variable power cycling (40–140% of maximal aerobic power or MAP) was performed than cycling at a constant power output (65% of MAP) [27]. These effects were also observable in the swimming segment, showing a decrease in VO_2_max (−4%) as well as a decrease in maximal cycling power (−4.8%) when cycling was preceded by a 2 km swimming test and when it was compared to an isolated cycling test [32]. Additionally, a significant increase in VE, FR, HR, and VO_2_ was observed during cycling when the swimming segment previously performed was at a greater intensity.

- Training program.

Several studies analyzed the effects of different training programs carried out by triathletes during a specific period of time [1,20,24,26,28]. These trainings were classified into three zones according to the intensity distribution: Zone 1 (Z1) corresponds to low intensity exercise (at or below VT_1_), Zone 2 (Z2) corresponds to moderate intensity (between VT_1_ and VT_2_), and Zone 3 (Z3) corresponds to high intensity exercise (at or beyond VT_2_) [1,20]. Thus, in a study made up of recreational-level triathletes divided in two groups that underwent a 13-week polarized training (80% in Z1) and threshold training (78% in Z1), they showed a significant increase in VO_2_max and maximal aerobic power and speed in cycling and running [20]. However, in another study in which a 12-week threshold training (64% in Z1) with a higher percentage of moderate intensity training was performed, no physiological improvements were observed in cycling and running [1].

Other authors have compared the effectiveness of high-volume and low-intensity training (Z1) with low-volume and high-intensity interval training or HIIT (Z3) performed by a group of triathletes for 4 weeks. The results showed significant improvements in VO_2_max (+6.7%) in the HIIT group, as well as improvements in aerobic power and speed of cycling and running, but only in the low-intensity training group (170 W vs. 183 W and 29.4 min vs. 27.1 min, respectively) [26]. In this way, all training programs improved triathletes’ performance, where cycling and running were the segments that showed the best results [3,19,32]. These improvements were related to an increase in VO_2_max and maximal aerobic power [1,3,19], as well as a decrease in triathletes’ body composition [32].

- Triathletes’ performance related to other athletes.

One article compared the differences between physiological variables in triathletes and athletes who practiced other sports [19]. It was observed that there were no significant differences in VO_2_max levels between elite triathletes and elite soccer players. Therefore, the results obtained were considered inconclusive.

### 3.5. Data Synthesis and Meta-Analysis

**Figure 4:**
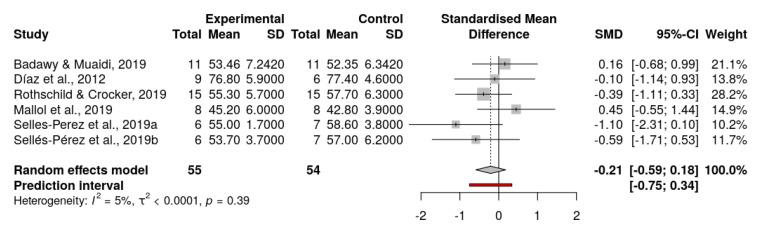
Forest plot [6,19,20,21,24,26].

To carry out the meta-analysis, the articles were grouped so that they had at least two intervention groups. Primary outcome measures, related to VO_2_max levels, were obtained and the meta-analysis was performed. After collecting data from the studies included in the meta-analysis (Table 7), the results obtained were analyzed. These results determined the mean effect size obtained in a sample of 109 athletes. Thus, a standardized mean difference of SMD = −0.21 was observed, with a confidence interval of −0.84 to 0.43. Therefore, no significant differences between the experimental group and the control group in terms of VO_2_max were found (Figure 4).

Regarding the degree of heterogeneity of the effect size of the studies, an I^2^ = 5% variability index was observed, which determines a low degree of heterogeneity. Furthermore, a *p* = 0.39 value was obtained, which means that the results are considered non-statistically significant. Therefore, based on the preliminary results, we cannot establish conclusive results regarding the effect of VO_2_max changes on triathletes’ performance.

## 4. Discussion

The objective of this systematic review and meta-analysis was to investigate the changes produced in cardiorespiratory fitness that determine the level of triathletes’ performance, measured through VO_2_max. After performing the analysis of the articles selected, some considerations about the studies included in this paper need to be added.

### 4.1. Cardiorespiratory Fitness in Triathletes

These physiological parameters usually change depending on certain factors, such as the triathlon segment performed, the competition distance, and the triathlete’s age and sex.

#### 4.1.1. Physiological Response Depending on the Competition Segment

The execution of the cycling segment can negatively affect subsequent running performance due to increases in triathletes’ cardiorespiratory fitness during running [3,22,23,25,26]. This increase in cardiorespiratory fitness is related to the execution of a high intensity cycling segment, which produces higher energy consumption (ATP) that results in the accumulation of protons, leading to a more acidic environment and a subsequent increase in ventilation and in respiratory fatigue [3,27,36,37]. This energy consumption also increases blood lactate concentration, so increases in this value could be considered as an indirect indicator of metabolic acidosis. A decrease in VO_2_max was also observed, which would make sports recovery between high intensity efforts more difficult [3,27,29,31,33]. Additionally, this increase in cardiorespiratory fitness is associated with changes in muscle recruitment of the lower limbs, which triggered biomechanical alterations, such as a decrease in stride length and an increase in its frequency, as well as decreases in maximal aerobic speed during running [22,31,32]. However, these changes were not observable in elite athletes, which suggests that more trained triathletes tend to have fewer alterations in sports performance than less experienced triathletes [21,29].

As with cycling, swimming also affects the performance of the following segment, with increases observed in cardiorespiratory fitness during cycling, especially when the swimming segment was performed at a higher intensity. These effects could be a result of an increase in respiratory muscle fatigue during this segment, due to the fact that higher intensity work was performed by the respiratory muscles during swimming [35].

#### 4.1.2. Physiological Response Depending on Competitive Distance, Age, and Sex

There are other factors that determine triathletes’ performance, such as the competition distance and the athlete’s age and sex. In this way, as the competition distance increases, triathletes achieve their best race performance at an older age [7]. It was observed that the ages of participants’ highest performance tend to be ~27, ~30, and ~33 years old for the Olympic, half-Ironman, and Ironman distances respectively [2,7]. Therefore, age is a predictive variable of triathlon performance. This performance is relatively stable until 35–40 years of age and decreases progressively after 50 years old, especially in women [7,27,34].

Although gender differences in triathlon performance have decreased in the last three decades, both in recreational-level and elite triathletes, there are other differences in triathletes’ performance depending on the triathlon segment performed [8]. These differences were lesser in swimming, since women perform better, possibly because they have a higher body fat percentage than men, providing greater buoyancy and better performance in this segment. In addition, men tend to have a higher muscle mass percentage, which is associated with greater cycling and running power output, which leads to improvements in the performance of these segments. Women also tend to have lower VO_2_max values than men, which are associated with lower maximal power and speed in cycling and running [2,8].

### 4.2. Implication for Sports Practice

Keeping in mind that the performance can be affected depending on the athletes’ physiological responses, it is necessary for triathletes to train specifically to improve their performance in competitions. Based on the results, high-volume and low-intensity training (Z1) seems to produce greater physiological adaptations in cycling and running than moderate-intensity training (Z2), possibly because it allows better sports recovery between training sessions, minimizing accumulated fatigue and improving performance in both segments [1,20,24,28]. Meanwhile, high intensity training seems to produce increases in athletes’ VO_2_max, which is also associated with improvements in cycling and running power and speed [24]. Therefore, high intensity training improved triathletes’ aerobic endurance, while low-intensity training improved their performance. These improvements were only observed in cycling and running, as both segments benefited from so-called cross training [1,24,28]. The effects of cross training were not shown in the swimming segment, perhaps due to the highly technical component of this sport compared to cycling and running [1,20,35]. Swimming requires more training than the other two segments, with focusing on swimming technique improving performance of this segment [1,35].

Some limitations of this study need to be addressed. First, the studies were heterogeneous, making comparisons difficult. For this reason, out of the twenty-five articles included in the review, only six provided information to the meta-analysis. Consequently, it was not possible to carry out a sub-analysis based on age, triathlon segment execution, or training program. All this makes statistical comparison of VO_2_max changes difficult. Moreover, studies selected for this paper have a low methodological quality since articles that met the inclusion criteria were not found. Future research that incorporates studies with higher methodological quality is necessary to evaluate those factors that influence cardiorespiratory fitness in triathletes during sports practice. It is also recommended to carry out studies that focus on analyzing populations of elite triathletes who have competed in sprint distances to measure the changes produced in cardiorespiratory variables in this population.

## 5. Conclusions

Based on the results obtained, it can be concluded that cardiorespiratory fitness, mainly in terms of VO_2_max, is a strong predictor of sports performance in triathletes. Triathletes tend to have higher VO_2_max values, although this response may vary depending on the age, gender, and triathlon segment. In all cases, specific training of the different triathlon segments can generate physiological adaptations that allow athletes’ aerobic endurance and competition performance to be improved, especially if low-intensity and high-volume training is combined with high-intensity interval training.

## Figures and Tables

**Table 1:** Conventional triathlon formats.

Format	Swimming	Cycling	Running	Distance
Super-sprint	400 m	10 km	2.5 km	Short
Sprint *	750 m	20 km	5 km	Short
Olympic *	1.5 km	40 km	10 km	Half
Half distance/half-Ironman/70.3 *	1.9 km	90 km	21 km	Long
Ironman™ *	3.8 km	180 km	42 km	Long
Long-distance	4 km	120 km	30 km	Long

* Determines the distances studied in the present study.

**Table 2:** Search strategy.

Databases	Total Articles Found	Search
PubMed	127	(VO_2_max) OR (VO_2_peak) OR (maximal oxygen uptake) AND (triathlon) (ventilatory thresholds) OR (aerobic thresholds) AND (triathlon) (cardiorespiratory response) OR (cardiorespiratory fitness) AND (triathlon) (physiological variables) AND (triathlon)
SPORTDiscus	142	
Embase	77	
Web of Science	235	
MEDLINE	114	
Dialnet	18	(VO_2_max) AND (triathlon) (ventilatory thresholds) AND (triathlon) (respuesta cardiorrespiratoria) AND (triatlón) (physiological variables) AND (triathlon)

**Table 3:** PEDro scale.

Study	Total Score	1	2	3	4	5	6	7	8	9	10	11
Badawy & Muaidi, 2019 [19]	4/10	–			×				×	×	×	
Díaz et al., 2012 [21]	5/10	–	×		×				×	×	×	
Olcina et al., 2019 [22]	3/10	–	×		×							×
Sultana et al., 2012 [23]	7/10	–			×		×	×	×	×	×	×
Sellés-Pérez et al., 2019 [24]	3/10	–			×				×	×		
Peiffer et al., 2016 [25]	4/10	–			×				×	×	×	
Rothschild & Crocker, 2019 [6]	3/10	–			×				×			×
Mallol et al., 2019 [26]	5/10	–	×		×				×		×	×
Etxebarria, Anson, et al., 2013 [3]	5/10	–	×		×				×	×		×
Selles-Perez et al., 2019 [20]	3/10	–			×						×	×
Etxebarria, Hunt, et al., 2013 [27]	4/10	–			×				×	×		×
Etxebarria, Anson et al., 2013 [28]	5/10	–	×		×				×	×		×
Walsh et al., 2015 [29]	4/10	–			×				×	×		×
Rodríguez-González & Rodríguez-Marroyo, 2017 [1]	4/10	–			×				×	×		×
Lorenzo Capellá et al., 2018 [30]	7/10	–			×	×	×	×	×	×		×
Walsh et al., 2017 [31]	3/10	–			×				×	×		

The symbol “×” indicates those items that have been scored; the symbol “–“ indicates those items that were not counted for the final score.

**Table 4:** AMSTAR-2 scale.

Study	Items																	
	1	2	3	4	5	6	7	8	9	10	11	12	13	14	15	16	Total	RQ
Walsh, 2019 [32]								×		×	–	–		×		×	4/14	CL
Lepers, 2019 [8]	×									×	–	–		×		×	4/14	CL
Millet et al., 2011 [33]	×										–	–					1/14	CL
Suriano & Bishop, 2010 [5]	×										–	–					1/14	CL
Knechtle et al., 2015 [7]	×										–	–				×	2/14	CL
Lepers et al., 2013 [2]	×									×	–	–				×	3/14	CL
Etter et al., 2013 [34]	×										–	–					1/14	CL
Cuba-Dorado & García-García, 2014 [4]	×										–	–					1/14	CL
Olbrecht, 2011 [35]	×										–	–	×				2/14	CL

The symbol “×” indicates those items that have been scored; the symbol “–“ indicates those items that were not counted for the final score. I, item; RQ, review quality; CL, critically low.

**Table 5:** PICO Table.

Study	Sample Design	Intervention	Duration	Outcome Measure	Measuring Instrument	Results
Badawy & Muaidi, 2019 [19]	Cohort study (*N* = 22) B quality	- EX: modified Bruce protocol - CON: standard Bruce protocol	2 sessions	VO_2_max, HRmax, RPE, height, body mass, and body fat	Exercise stress tests with gas analyzer HR monitor Borg 0–10 Scale Body composition analyzer	There were no significant differences in absolute and relative VO_2_max values, nor in HRmax, between soccer players and triathletes (*p* > 0.05). However, significant increases in relative and absolute VO_2_max (*p* < 0.05) were obtained in triathletes and soccer players in EX compared to CON, which were significantly correlated with the duration of the test (r = 0.534 y r = 0.563 respectively). Furthermore, there were no significant changes in HRmax (*p* > 0.05). Therefore, maximum values were able to be obtained without leading to excessive cardiovascular stress during the test.
Díaz et al., 2012 [21]	Cohort study (*N* = 15) B quality	- EX: 30-min cycling test and 3 km running test - CON: 3 km running test	2 seasons	VO_2_max, ventilatory thresholds, VE, VE/VO_2_, VE/VCO_2_, RR, HR, RPE, and speed (distance per time)	Maximal exercise stress tests with gas analyzer HR monitor Borg 0–10 Scale Chronometer	In the TID group, there was a significant increase in cardiorespiratory fitness and HR in EX compared to CON (*p* < 0.01) in both seasons. Nevertheless, no changes related to this response were found in the SE group. Furthermore, in both seasons, the completion of the time-trial run was significantly longer in EX than in CON, both in TID (*p* < 0.01) and in the SE group (*p* < 0.05).
Olcina et al., 2019 [22]	Randomized controlled trial (*N* = 10) B quality	- EX: 20-min cycling test and 12-min running test - CON: 12-min running test	2 sessions	%SmO_2_, HR, RPE, pain, running power and kinematics, height, and body mass	Maximal exercise stress tests with NIRS. Time-trial cycling test with NIRS Borg 6–20 Scale VAS pain 0–10 Body composition analyzer	The results showed that running distance (ES = 0.6; *p* = 0.00) and stride length (ES = 0.4; *p* = 0.00) were significantly lower in EX than in CON. On the other hand, %SmO_2_ was significantly lower in CON than in EX (ES = 1.63; *p* < 0.01), which makes it the most modified value by previous cycling performance.
Sultana et al., 2012 [23]	Cohort study (*N* = 19) C quality	- EX1: Olympic distance triathlon performed by master triathletes - EX2: Olympic distance triathlon performed by young triathletes	3 sessions	VO_2_max, ventilatory thresholds, running economy, HR, running speed, RPE, body mass, and volume of fluid ingested	Maximal exercise stress tests with gas analyzer. HR monitor Borg 6–20 Scale Chronometer Isometric dynamometer Questionnaire of the volume of fluid ingested	During pre-test, VO_2_max and ventilatory thresholds were significantly lower in EX compared to CON (r = 0.76), with a decrease in these values in the post-test for both groups (*p* < 0.05). Similarly, in the pre-test, running economy was significantly higher (+5.2%) in EX compared to CON. There were no significant differences in maximal isometric torque in both groups (*p* > 0.05).
Sellés-Pérez et al., 2019 [24]	Cohort study (*N* = 14) C quality	- EX: 20-week specific polarized training program (in mesocycles)	20-week	VO_2_max, ventilatory thresholds, cycling and running power and speed, swimming speed, height, body mass, body fat, and skinfolds	Maximal exercise stress tests with gas analyzer Chronometer Skinfold caliper, stadiometer, and anthropometric tape	Training improved most of the variables related to sports performance, especially in triathletes with a lower initial performance level. A significant increase in VO_2_max was observed in cycling (*p* < 0.05) and running (*p* < 0.01) in most triathletes when compared to the pre-test, mostly in those who were endomorphic (ρ = 0.716; *p* < 0.01). In addition, a decrease in body composition was observed.
Rothschild & Crocker, 2019 [6]	Cohort study (*N* = 15) C Quality	- EX: 2 km swimming test and an incremental cycling test - CON: an incremental cycling test	2 sessions	VO_2_max, HR, blood lactate concentration, cycling power output, body mass, body fat, and hydration changes	Maximal and submaximal exercise stress tests with gas analyzer HR monitor Blood lactate analyzer Bioelectrical impedance analyzer Weighing scale	The study showed that 2 km swimming in EX produced a significant decrease in VO_2_max (*p* = 0.01), maximal cycling power output (*p* < 0.01) and cycling power at the lactate threshold (*p* = 0.03), as well as a significant increase in submaximal HR (*p* = 0.02) when compared to CON.
Mallol et al., 2019 [26]	Cohort study (*N* = 16) B Quality	- EX: low-volume and high-intensity training and a cycle-run sprint triathlon simulation - CON: usual high-volume training and a cycle-run sprint triathlon simulation	4 weeks	VO_2_max, HR, cycling power output in VT_1_ and VT_2_, RPE, running speed, height, and body mass	Maximal and submaximal exercise stress tests with gas analyzer HR monitor Borg 0–10 Scale	There was a significant increase in VO_2_max (ES = 0.5; *p* < 0.05) and in cycling power output at VT_1_ and VT_2_ (*p* = 0.03) in EX compared to CON after the incremental test. However, after the triathlon simulation, there were no significant changes in performance variables in the EX group, but there was an increase in running performance in the CON group (ES = 0.53; *p* = 0.04).
Etxebarria, Anson, et al., 2013 [3]	Cohort study (*N* = 12) B Quality	- EX1: 1 h cycling test at a variable power output and 9.3 km running test - EX2: 1 h cycling test at a constant power output and 9.3 km running test - CON: 9.3 km running test	4 sessions	VO_2_max, maximal cycling power, blood lactate concentration, HR, RPE, height, body mass, skinfolds, and hydration status	Maximal exercise stress tests with indirect calorimetry system Blood lactate analyzer HR monitor Borg 0–10 Scale Digital urine refractometer	The results showed that running performance in CON was higher than in EX1 (r = 0.63) and EX2 (r = 0.40). Furthermore, running performance after EX1 was lower than after EX2 (r = 0.21). These values were related to a greater increase in blood lactate concentration (r = 0.51) and RPE (r = 0.55) after EX1 when compared to EX2 values.
Selles-Perez et al., 2019 [20]	Cohort study (*N* = 18) C Quality	- EX: polarized triathlon training - CON: pyramidal triathlon training	20 weeks	VO_2_max, ventilatory thresholds, HR, RPE, cycling power output and swimming, and running speed	Maximal exercise stress tests with gas analyzer HR monitor Borg 0–10 Scale	A significant increase in the performance of the three segments was found in EX and CON (*p* < 0.05), without significant differences between both groups. However, an improvement in triathlon performance was found when triathletes trained for a longer time between VT_1_ and VT_2_, and a performance decrease was found when they trained for longer at VT_2_ (*p* < 0.05).
Etxebarria, Hunt, et al., 2013 [27]	Cohort study (*N* = 9) C Quality	- EX1: 1 h cycling variable power output test and a submaximal incremental running test - EX2: 1 h cycling constant power output test and a submaximal incremental running test - CON: submaximal incremental running test	4 sessions	VO_2_max, VO_2_, VCO_2_, VE, blood lactate concentrate, HR, cycling power output, RPE, and body mass	Maximal exercise stress tests with gas analyzer Blood lactate analyzer HR monitor Borg 6–20 Scale Electronic scale	The results showed that there was greater physiological demand in EX1 compared to EX2 due to the fact that a significant increase in VE (ES = 1.2; *p* = 0.02), VE/VO_2_ (ES = 1.2; *p* = 0.00), and blood lactate concentration (ES = 2.1; *p* = 0.00) during EX1 was observed when compared to EX2. Similarly, there was a significant increase in VE (ES = 1.2) and blood lactate concentrate (ES = 2.1), HR (ES = 1.1) and RPE (ES = 1.3) (*p* < 0.05) during running after EX1 and EX2 compared to CON.
Etxebarria, Anson, et al., 2013 [28]	Cohort study (*N* = 14) B Quality	- EX: short high-intensity interval training - CON: long high-intensity interval training	6 sessions	VO_2_max, maximal cycling power output, blood lactate concentration, HR, RPE, height, and body mass	Maximal exercise stress tests with indirect calorimetry system Blood lactate analyzer HR monitor Borg 0–10 Scale	The results concluded that both EX and CON induced physiological improvements in triathletes, producing a small–moderate increase in VO_2_max (+7%) and maximal aerobic power (+6%). These changes made it possible to generate adaptations to high intensity efforts in cycling, substantially improving subsequent running time, mainly in EX (67%).
Walsh et al., 2015 [29]	Cohort study (*N* = 6) C Quality	- EX: 20 min variable power cycling test and 30-min running test - CON: 10 min running test	1 session	VO_2_, HR, and muscle recruitment activity	Submaximal exercise stress tests with gas analyzer HR monitor Electromyogram (EMG)	The results showed no differences in EMG activity between EX and CON. However, a significant increase in VO_2_ (*p* = 0.02) and in HR (*p* < 0.01) was obtained at the beginning of the running test in EX, but without any difference at the end of it. Therefore, there was no correlation between muscle recruitment and VO_2_ in post-cycling running performance.
Rodríguez-González & Rodríguez-Marroyo, 2017 [1]	Cohort study (*N* = 20) C Quality	- EX: 12 week threshold training	12 weeks	VO_2_max, VO_2_, VCO_2_, ventilatory thresholds, blood lactate concentration, HR, RPE, cycling power output, running economy, and speed	Maximal and submaximal exercise stress tests with gas analyzer Blood lactate analyzer HR monitor Borg 0–10 Scale Chronometer	Significant improvements were obtained in cycling and running performance, with a significant increase in maximal speed and in speed at VT_2_ observed for running (*p* < 0.05), as well as an increase in VO_2_ and %VO_2_max at VT_1_ and VT_2_, and in maximal power output and in power output at VT_1_ and VT_2_ for cycling (*p* < 0.05). However, non-significant changes were observed in running economy or swimming performance (*p* > 0.05).
Walsh et al., 2017 [31]	Cohort study (*N* = 8) C Quality	- EX: 20 min variable power cycling test and 30 min running test - CON: 10 min running test	1 session	VO_2_, VCO_2_, VE, VE/VO_2_, VE/VCO_2_, RR, RER, HR, running economy and kinematics, RPE, height, and body mass	Submaximal exercise stress tests with gas analyzer HR monitor Motion capture analyzer Borg 6–20 Scale Stadiometer and anthropometric tape	There was a significant increase in VE, VE/VCO_2_, RER, and RR (*p* = 0.01), and in HR (*p* < 0.05) during running in EX compared to CON, as well as a significant increase in mean VO_2_ and in running economy at the beginning of the running segment. In addition, there was a significant increase in stride frequency, such as a decrease in stride length, in EX compared to CON (*p* = 0.01).

*N*: sample size; EX: experimental group; CON: control group; RPE: rating of perceived exertion; r: Pearson’s correlation coefficient; TID: talent identification triathlete group (highly-trained); SE: senior elite triathlete group; NIRS: near-infrared spectroscopy; ES: effect size; VT_1_: first ventilatory threshold; VT_2_: second ventilatory threshold.

**Table 6:** Study variables.

Study	VO_2_max	VTs	Ventilatory Measures	Blood Lactate Concentration	HR	Anthropometric Measures	Physiological Measures	Perceived Measures	Speed and Power Output	Hydration
Badawy & Muaidi, 2019 [19]	×				×	×		×		
Díaz et al., 2012 [21]	×	×	×		×			×	×	
Olcina et al., 2019 [22]			×		×		×	×	×	
Sultana et al., 2012 [23]	×	×			×	×		×	×	×
Sellés-Pérez et al., 2019 [24]	×	×				×			×	
Rothschild & Crocker, 2019 [6]	×			×	×	×			×	×
Mallol et al. 2019 [26]	×	×			×	×		×	×	
Etxebarria, Anson, et al., 2013 [3]	×			×	×	×		×	×	×
Selles-Perez et al., 2019 [20]	×	×			×			×	×	
Etxebarria, Hunt, et al. 2013 [27]			×	×	×	×		×	×	
Etxebarria, Anson, et al., 2013 [28]	×			×	×	×		×	×	
Walsh et al., 2015 [29]			×		×		×			
Rodríguez-González & Rodríguez-Marroyo, 2017 [1]	×	×	×	×	×		×	×		
Walsh et al., 2017 [31]			×		×	×	×	×		

The symbol “×” indicates those items that have been scored. Ventilatory measures include VO_2_ (mL/min/kg), VCO_2_ (mL/min/kg), VE (L/min), RR (breaths/min), VE/VO_2_, VE/VCO_2_, RR, and/or %SmO_2_; Anthropometric measures include: age (years), height (cm), weight (kg); HR (beats/min); speed (km/h); power output (W/kg).

**Table 7:** Data removed from the studies included in the meta-analysis.

Study	*N* EX	Mean EX	SD EX	*N* CON	Mean CON	SD CON
Badawy & Muaidi, 2019 [19]	11	53.46	7.242	11	52.35	6.342
Díaz et al., 2012 [21]	9	76.8	5.9	6	77.4	4.6
Rothschild & Crocker, 2019 [6]	15	55.3	5.7	15	57.7	6.3
Mallol et al., 2019 [26]	8	45.2	6	8	42.8	3.9
Sellés-Pérez et al., 2019 [24]	6	53.7	3.7	7	57	6.2
Selles-Perez et al., 2019 [20]	6	55	1.7	7	58.6	3.8

Maximal oxygen consumption (VO_2_max) expressed in mL/kg/min. *N*: sample size; SD: standard deviation.
